# Facial pain of cardiac origin: a case report

**DOI:** 10.1590/S1516-31802006000300012

**Published:** 2006-05-04

**Authors:** Ana Carolina de Oliveira Franco, José Tadeu Tesseroli de Siqueira, Alfredo José Mansur

**Keywords:** Facial pain, Temporomandibular joint disorders, Angina pectoris, Myocardial infarction, Dor orofacial, Transtornos da articulação temporomandibular, Angina pectoris, Infarto do miocárdio, Cardiopatia isquêmica

## Abstract

**CONTEXT:**

Cardiac pain may radiate to the face and lead patients to seek dental care. Dentists may contribute towards the diagnosing of ischemic heart disease and thus refer patients for cardiological evaluation.

**CASE REPORT:**

A 50-year-old female patient was referred to a dentist for evaluation of a suspected temporomandibular disorder after repeated visits to medical emergency departments due to excruciating facial and left temporal pain associated with exertion. The pain would start in the chest and radiate to the neck, face and left temporal region. The patient's chief complaint was the facial pain; hence, she sought dental care. The dental examination revealed an edentulous upper jaw and partially edentulous lower jaw with full upper prosthetic set of teeth and decreased vertical dimension. X-ray of facial bones did not reveal any bone abnormalities. A diagnosis of temporomandibular disorder was made. However, she was referred for cardiological evaluation, since her pain was starting in the chest and because she had a past medical history of surgical treatment for coronary artery disease. A diagnosis of angina pectoris was made, the therapeutic regimen was optimized and her angina was brought under control.

## RESUMO

**CONTEXTO:**

A dor de origem cardíaca pode apresentar irradiação para a face e motivar pacientes a procurarem atendimento odontológico. Nestas situações, cirurgiões-dentistas podem contribuir para o diagnóstico da cardiopatia isquêmica e serem encaminhadores de pacientes para avaliação cardiológica.

**RELATO DE CASO:**

Mulher de 50 anos de idade, portadora de diabetes melito, foi encaminhada para avaliação odontológica de disfunção temporomandibular, depois de repetidas consultas em serviço de emergência em razão da dor facial e bitemporal associada ao esforço físico. A dor iniciava-se no tórax, irradiava para a região cervical, face e região temporal à esquerda há seis meses. Embora houvesse dor torácica, a queixa principal da paciente era de dor facial, o que a motivou à procura de atendimento odontológico. O exame odontológico revelou paciente edêntula na parte superior e desdentada parcial na inferior da boca, em uso de prótese total superior com perda da dimensão vertical. O exame radiográfico da face não mostrou anormalidades ósseas. Foi feito diagnóstico de disfunção temporomandibular, mas, tendo em vista a dor torácica e o antecedente de cardiopatia isquêmica operada, a paciente foi encaminhada para avaliação cardiológica. Foi feito o diagnóstico de angina do peito e o quadro foi controlado com o ajuste do tratamento medicamentoso.

## INTRODUCTION

Complaints of pain in the face or teeth have been recognized as a symptom of angina pectoris or myocardial infarction.^1^ There are reports of facial pain caused by ischemic heart disease. Cases of misdiagnosis that led to unnecessary dental treatment such as endodontic procedures have been reported.^2^ We report the case of a patient whose chief complaint was excruciating facial pain that, after deeper investigation, was attributed to angina pectoris due to ischemic heart disease.

## CASE REPORT

A 50-year-old diabetic woman with the chief complaint of excruciating pain in the upper and lower jaw and left temporal area that had lasted for six months was referred to a dentist for evaluation of a suspected temporomandibular disorder. The pain was severe to the point of repeated visits to medical emergency departments.

On evaluation, she said that the pain would start in the chest and radiate to the neck, face and left temporal region. These five-minute bouts of pain would occur every day, triggered by exertion and emotions, and they were alleviated by resting. She also complained of pain in masticatory muscles during mastication; nonsteroidal anti-inflammatory drugs alleviated this pain.

The dental examination revealed an edentulous upper jaw with a full prosthetic set of teeth and partially edentulous lower jaw and decreased vertical dimension. Physical examination revealed pain not only in masseter but also in pterygoid muscles. X-ray of facial bones did not reveal any bone abnormalities. A diagnosis of temporomandibular disorder was made. Upper and lower appliances were installed for vertical dimension repositioning, and the patient was freed from 50% of her facial pain.

She was eventually referred for cardiological evaluation, since her pain was starting in the chest and because she had a past medical history of coronary artery bypass grafting surgery, some years previously. A diagnosis of angina pectoris was made and proper treatment was introduced. The therapy was successful and the patient became free of angina after nine months.

## DISCUSSION

The chief complaint of this patient was facial pain. This was the reason why she was first referred to a dentist for evaluation of a suspected temporomandibular disorder, in spite of her past medical history of coronary artery bypass graft surgery. Moreover, this first diagnosis was supported by the fact that the patient also complained of pain in masticatory muscles during mastication. Physical examination revealed pain in the mastica-tory system that led to the diagnosing of a temporomandibular disorder, and the correct treatment was instituted. However, the pain was associated with exertion and she was eventually referred for cardiological evaluation. The patient's dental condition was also poor, with lost teeth, which suggested a possible source of the facial pain. It cannot be overemphasized how important it is to have an accurate evaluation of the quality of the pain.

Several reports have demonstrated that improper diagnosis leads to unnecessary dental treatment.^1–3^ Indeed, there are reports of patients who underwent dental extractions for the treatment of facial pain that was eventually diagnosed as angina due to ischemic heart disease.^2^

Myofascial pain from masticatory muscles may be acute and regional, or chronic associated with widespread pain, and may have atypical presentation, such as angina. The masticatory system contributes towards a variety of clinical presentations and may further complicate diagnosis. There may be complaints of pain in the preauricular, face, jaw, temporal and occipital areas, and palpation of the masticatory muscles (masseter, pterygoid and temporal) on physical examination may increase the pain in these muscles.^4^ Loss of posterior occlusal support, due to missing teeth associated with loss of vertical dimension, may contribute towards musculoskeletal pain. These conditions may increase the risk of pain and dysfunction in susceptible individuals. Thus, elimination of the causal factors and the use of rehabilitative procedures are useful for eliminating pain.^4^

When facial pain is recognized as a possible sign of heart disease, the dentist may have an important role as a diagnostician. Careful history-taking regarding the pain is of paramount importance.^1–3^ In these settings, dentists may contribute towards the diagnosing of ischemic heart disease and thus refer patients for cardiological evaluation.
